# Progressively shifting patterns of co-modulation among premotor cortex neurons carry dynamically similar signals during action execution and observation

**DOI:** 10.1101/2023.11.06.565833

**Published:** 2023-11-06

**Authors:** Zhonghao Zhao, Marc H. Schieber

**Affiliations:** 1 Department of Biomedical Engineering, University of Rochester, Rochester, NY, 14627; 2 Department of Neurology, University of Rochester, Rochester, NY, 14642; 3 Department of Neuroscience, University of Rochester, Rochester, NY 14642

## Abstract

Many neurons in the premotor cortex show firing rate modulation whether the subject performs an action or observes another individual performing the same action. Although such “mirror neurons” have been thought to have highly congruent discharge during execution and observation, many if not most show non-congruent activity. Studies of such neuronal populations have shown that the most prevalent patterns of co-modulation—captured as neural trajectories—pass through subspaces which are shared in part, but in part are visited exclusively during either execution or observation. These studies focused on reaching movements for which the neural trajectories show comparatively simple dynamical motifs. But the neural dynamics of hand movements are more complex. We developed a novel approach to examine prevalent patterns of co-modulation during execution and observation of a task that involved reaching, grasping and manipulation. Rather than following neural trajectories in subspaces that contain their entire time course, we identified time series of instantaneous subspaces, sampled trajectory segments at the times of selected behavioral events, and projected each segment into the series of instantaneous subspaces. We found that instantaneous neural subspaces were partially shared between execution and observation in only one of three monkeys and were otherwise exclusive to one context or the other. Nevertheless, the patterns during execution and observation could be aligned with canonical correlation, indicating that though distinct, neural representations during execution and observation show dynamical similarity that may enable the nervous system to recognize particular actions whether performed by the subject or by another individual.

## INTRODUCTION

Although the premotor (PM) and primary motor cortex (M1) are generally thought to be involved in the planning and execution of movement, many neurons in these cortical motor areas have been found to discharge not only when the subject executes a movement, but also when the subject observes a similar movement being performed by another individual. Such neurons have been found in the ventral premotor cortex (PMv) (Bonini et al., 2014; Gallese et al., 1996), dorsal premotor cortex (PMd) (Cisek & Kalaska, 2004; Papadourakis & Raos, 2019), and M1 (Dushanova & Donoghue, 2010; Kraskov et al., 2014a; Vigneswaran et al., 2013).

Early studies of these neurons emphasized those with “congruent” discharge during execution and observation conditions. Congruent neurons discharged during the same type of grasp (Gallese et al., 1996; Rizzolatti et al., 1996), or retained the same preferred direction (Dushanova & Donoghue, 2010; Kilner & Lemon, 2013) during both execution and observation. Emphasis on such congruent neurons led to the notion that they mediate understanding of observed actions by mirroring their own activity during execution (di Pellegrino et al., 1992; Rizzolatti and Craighero, 2004).

Even early studies also reported, however, many other “noncongruent” neurons that also discharged during execution and during observation, but differently in the two contexts (Gallese et al., 1996). For example, of the pyramidal tract neurons (PTNs) in PMv and M1 that show modulation during both execution and observation, half show substantially lower firing rates during observation than during execution (Kraskov et al., 2009; Vigneswaran et al., 2013; Kraskov et al., 2014). In many studies roughly half or more of the neurons modulated during both execution and observation are noncongruent (Dushanova and Donoghue, 2010; Kraskov et al., 2014; Mazurek et al., 2018; Jiang et al., 2020). Of PMv neurons modulated during both execution and observation, over the time course of behavioral trials only ~20% showed brief periods with strictly congruent firing rates (Pomper et al., 2023). And in PMd, the proportion of congruent MNs may not be different from that expected by chance alone (Papadourakis and Raos, 2019). That so many neurons are active differentially during action execution versus observation calls into question the extent to which the representation of movements by these neuron populations actually matches in the two contexts. Although many authors apply the term “mirror neurons” strictly to highly congruent neurons, like many others, we will refer to all neurons modulated during both contexts—execution and observation—as mirror neurons (MNs).

Addressing this issue at the population level is complex because of the wide variety of firing rate modulation found in individual neurons at different times in the course of behavioral trials, as well as among neurons in a recorded population. Different movements may be represented more accurately by the temporal evolution of co-modulation in populations of neurons than by the temporal pattern of discharge in single neurons. Patterns of co-modulation can be considered in a high-dimensional neural-state space where the firing rate of each neuron is a separate, orthogonal dimension. The instantaneous, simultaneous firing rates of all *N* neurons then is a point in this space, which traces out a trajectory over time. Neural population trajectories do not visit all regions of the *N*-dimensional state-space, however. Dimensionality reduction techniques can be used to identify a small set of latent dimensions—a subspace—that captures the most prevalent patterns of co-modulation among the population of *N* neurons. (Shenoy et al., 2013; Cunningham and Yu, 2014)

Studies of neural trajectories underlying action execution that focused on reaching movements made with the arm have revealing that rotational motifs in a low-dimensional subspace capture much of the neural population’s firing rate variance (Churchland et al., 2012). While similar rotational dynamics were found in M1 during repetitive cycling movements, the population trajectory of supplementary motor area neurons progressed as a helix through an additional dimension during successive cycles (Russo et al., 2020). The M1 neural trajectories underlying grasping movements made with the hand are still more complex, however (Suresh et al., 2020). The latent subspaces that capture the predominant patterns of condition-dependent co-modulation of M1 neurons during execution, for example, shift progressively over the time course of behavioral trials involving reaching to, grasping, and manipulating (RGM) various objects at various locations (Rouse and Schieber, 2018). Relatively few studies have examined the trajectories of neural populations that are active during both execution and observation (Mazurek et al., 2018; Jerjian et al., 2020; Jiang et al., 2020; Pezzulo et al., 2022).

**Figure 1.**
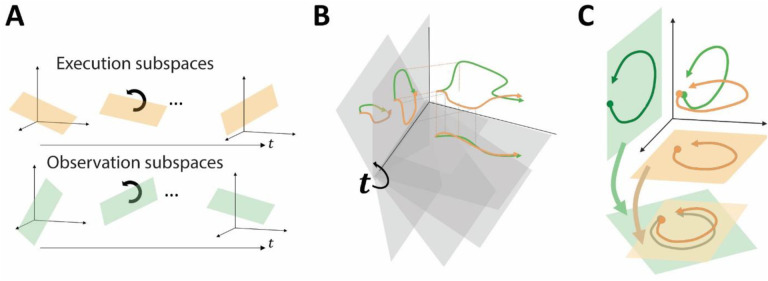
Conceptual approach. **A.** We hypothesized that the condition-dependent subspace of PM MN activity shifts progressively through the time course of behavioral trials both during execution (orange) and during observation (green). **B.** Trajectory segments (orange, green) were projected into time series of instantaneous subspaces (gray). **C.** Trajectory segments (latent dynamics) from execution and observation can be aligned even if they occupy distinct (orthogonal) subspaces.

In the present work, we examined the latent subspaces that capture the predominant patterns of co-modulation among populations of mirror neurons in the premotor cortex (PM MNs) during RGM movements, testing three hypotheses. First, we asked whether PM MN populations show progressive shifts in the latent dimensions (subspaces) that capture their condition-dependent patterns of co-modulation over the time course of both execution and observation trials, as illustrated schematically in Figure 1A. We developed a novel approach in which segments of the neural trajectories from behaviorally well-defined times are projected into the instantaneous subspaces at other times over the course of the same behavioral trials (Figure 1B). Because each of these subspaces is instantaneous, the projected trajectory segments capture condition-dependent variance in the neural activity while minimizing time-dependent (condition-independent) variance. We then evaluated the separation of the projected trajectory segments in each instantaneous subspace and the extent to which the segments could be decoded. We reasoned that the closer a given instantaneous subspace is to the subspace at the time from which the trajectory segments were taken, the greater the separation of trajectory segments representing different movements should be, and the better the decoding of those movements from the trajectory segments. Using this approach we examined the progressive evolution of the condition-dependent subspace over execution and over observation trials. We then asked whether the activity of the PM MN population progresses through similar subspaces during execution and observation. And third, we asked whether the execution and observation trajectory segments could be aligned (Figure 1C), indicating a corresponding structure in the distinct latent dynamic representations of execution and observation RGM movements by the same PM MN population.

## RESULTS

**Figure 2.**
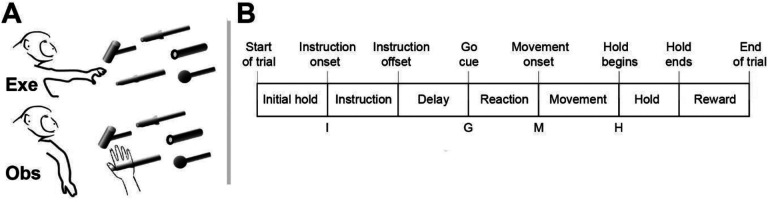
The reach-grasp-manipulate (RGM) task. **A.** In separate blocks of trials monkeys reached to, grasped, and manipulated four different objects themselves (Exe), and then observed a human perform the same task (Obs). **B.** The times of eight behavioral events from Start-of-trial to End-of-trial divided each trial into seven epochs from Initial hold to Reward. For analyses the data were aligned separately on, and trajectories were sampled at, the times of four selected events—Instruction onset (I), Go cue (G), Movement onset (M), and Hold (H).

We recorded spiking activity as each of three monkeys executed the RGM task, and then as each monkey observed the same RGM task being performed by an experimenter (Figure 2A). Additional details of the behavioral task are described in the Methods. Three sessions were recorded from each of the three monkeys, F, R, and T (a 10 kg male, 6 kg female, and 10 kg male, respectively). The numbers of successful execution trials (Exe) and observation trials (Obs) involving each of the four objects—sphere, button, coaxial cylinder, and perpendicular cylinder—are given in Table 1.

Using object and task time period as factors, we performed two-way ANOVA on the firing rate of each sorted unit (see Methods). Because unit firing rates almost always differed during execution and observation, we performed such ANOVAs separately on execution trials and observation trials. A unit was included for subsequent analysis if it showed significant firing rate modulation during both execution and observation. Although most studies have focused on neurons from either PMv or PMd, we combined units from these two cortical areas because neurons in each area have been shown to be modulated during both reaching and grasping (Stark et al., 2007). Table 2 gives the numbers of PM (PMv +PMd) units identified in each session as being modulated during both execution and observation.

### Instantaneous subspaces change during both execution and observation

Whereas a large fraction of condition-dependent neural variance during reaching movements without grasping can be captured by a two-dimensional subspace (Churchland et al., 2012; Ames et al., 2014), condition-dependent activity in movements that involve grasping evolves through more complex subspaces as the movements progress (Suresh et al., 2020). In part, this may reflect the greater complexity of controlling the 24 degrees of freedom in the hand and wrist as compared to the 4 in the elbow and shoulder (Sobinov and Bensmaia, 2021). Consistent with this complexity, over the time course of behavioral trials that entail concurrent reaching, grasping and manipulation (RGM), the condition-dependent activity of primary motor cortex (M1) neurons evolves through progressively changing neural subspaces (Rouse and Schieber, 2018).

Initially, we therefore asked whether the condition-dependent activity of mirror neurons in the premotor cortex likewise occupied shifting subspaces over the time course of RGM movements. To approach this question, for each recording session we applied PCA to identify the 2-dimensional subspace of condition-dependent activity at four behavioral time points readily defined across trials, sessions, and monkeys: onset of the instruction (I), go cue (G), onset of movement (M), and beginning of the hold (H). These subspaces consistently captured > 70% of the condition-dependent variance. (Note that because each subspace is instantaneous, no time-dependent variance is captured. And because here averaged across trials for each of the four target objects, noise variance was eliminated) Likewise for each session, we clipped four 100 ms segments of the high-dimensional PM mirror neuron population trial averaged trajectory beginning at the time of I, G, M, and H, for trials involving each of the four objects. We then projected each trajectory segment into each of these four instantaneous subspaces. This process was repeated separately for execution trials and for observation trials in each session. We reasoned that if the instantaneous subspace shifted little or not at all, then a given set of trajectory segments would have a similar projection into each instantaneous subspace, whereas if the subspace shifted these projections would change.

**Figure 3.**
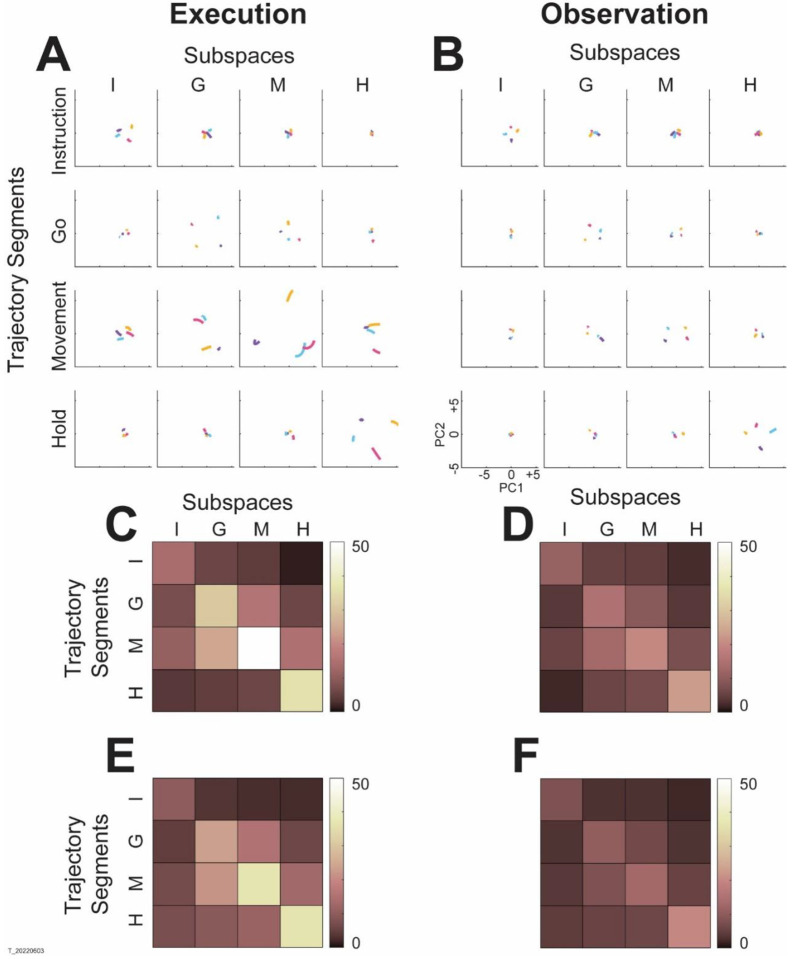
Trajectory segments projected into instantaneous subspaces. **A.** Using execution data from an example session (T_20220603), trajectory segments averaged across trials involving each of the four objects (sphere – purple, button – cyan, coaxial cylinder – magenta, perpendicular cylinder – yellow) were sampled immediately following each of four behavioral events (rows: Instruction onset, Go cue, Movement onset, Hold). Each set of these four segments then was projected into the instantaneous subspace present at four different times (columns: I, G, M, H). **B.** The same process was performed using observation data from the same session. The PC1 vs PC2 scales at lower left apply to all frames in both **A** and **B**. **C and D.** Cumulative separation values (CS, seem Methods) calculated for each of the frames in **A and B**, respectively, are shown as color matrices. **E** and **F** show CS values averaged across all 9 sessions for execution and observation, respectively.

Projections from an example session are illustrated in Figure 3. The trajectory segments for each of the four objects (sphere – purple, button – cyan, coaxial cylinder – magenta, perpendicular cylinder – yellow) sampled at different times (rows) have been projected into each of the four instantaneous subspaces (columns). Each set of trajectory segments thus is projected into its corresponding subspace along the main diagonal, showing that during execution (Figure 3A), the trajectory segments for the four objects are close together at the time of instruction onset (I), are more separated at the time of the go cue (G), have separated further still at movement onset (M), and are less separated at the time of the hold (H). Off-diagonal frames along the rows (same trajectory segments, different instantaneous subspaces) show less separation, indicating that each set of trajectory segments was most separated at the time near which it was clipped. Along the columns (different trajectory segments, same instantaneous subspaces), off-diagonal trajectory segments likewise show less separation, again indicating that each instantaneous subspace provided the greatest separation of the corresponding trajectory segments. These changes indicate that the condition-dependent subspace of this PM MN population changed as execution trials progressed in time, as illustrated schematically in Figure 1A.

During observation trials from the same session (Figure 3B), projections of the trajectory segments showed similar changes. In general less separation was evident for observation than for execution, reflecting the commonly described lower firing rates of MNs during observation (Ferroni et al., 2021). Nevertheless, projecting each set of trajectory segments into the instantaneous subspace at the corresponding time (main diagonal) provided the greatest separation. In contrast to execution, where the greatest separation was obtained for the Movement trajectory segments projected into the M subspace, for observation the Hold segments projected into the H subspace showed the greatest separation. Overall, however, the condition-dependent subspace of the PM MN population also changed as observation trials progressed in time, as illustrated schematically in Figure 1A.

To quantify these changes in the separation of the trajectory segments from trials involving the four different objects, we calculated the cumulative separation (C*S*, the summed pointwise distance between all pairwise combinations of the four object trajectory segments, see Methods) for each set of four segments projected into each of the four instantaneous subspaces. *CS* values of for execution are illustrated in the color matrix of Figure 3C; for observation, Figure 3D. For both execution and observation, the highest *CS* values lie on the main diagonal, increasing in temporal order from Instruction to Go to Movement to Hold, with the exception that for execution the separation for Hold was less than for Movement. Figure 3E and 3F show averaged CS matrices across all sessions from the three monkeys, demonstrating that the features seen in the example session were relatively consistent across sessions. During both execution and observation, the instantaneous subspace of the PM MN population changes over the time course of the behavioral trials.

### Instantaneous subspaces shift progressively through the time series of execution trials

We developed a novel approach to evaluate the instantaneous subspace shifting on a more continuous basis. For each session we first identified the instantaneous 3-dimensional neural subspaces at 50 ms intervals throughout the time course of execution trials. We then projected the Instruction, Go, Movement, and Hold trajectory segments from individual trials involving four objects into each of the instantaneous 3D subspaces in the time series. At each time point, we trained a separate LSTM decoder to classify individual trials according to which of the four objects was involved in that trial. We expected that the projection of trajectory segments would be decoded best near the time at which the trajectory segments were sampled. We reasoned that if at other times a given instantaneous subspace was very similar to that found at the time from which the segments were sampled, then the projection of the segments into that instantaneous subspace would be decoded almost as well. But if the other instantaneous subspace was dissimilar, then the projection into that subspace would be decoded poorly.

**Figure 4.**
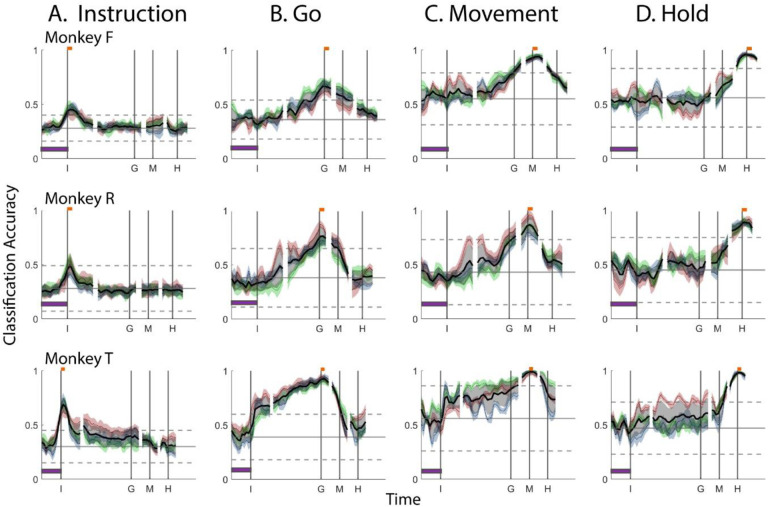
Classification accuracy for *execution trajectory segments* projected into instantaneous *execution subspaces*. **A.** Instruction trajectory segments. **B.** Go segments. **C.** Movement segments. **D.** Hold segments. In each frame, the short horizontal orange bars at the top of the vertical lines indicate the 100 ms during which each set of trajectory segments was sampled; the horizontal purple bar at lower left represents 500 ms. Results in 50 ms steps have been aligned separated at the times of the instruction onset (I), go cue (G), movement onset (M), and hold (H). Solid curves indicate mean classification accuracy of 10-fold cross-validation as a function of time, with the shaded areas indicating 1 standard deviation. Colors red, green, and blue represent sessions 1, 2, and 3 from each monkey, with black being their average. Horizontal black lines indicate the mean (solid) ± 3 standard deviations (dashed) classification accuracy obtained by projecting each set of trajectory segments into 500 randomly selected 3D spaces.

Figure 4 shows the resulting classification accuracy as a function of trial time for the 100 ms execution segments that were sampled beginning at the times of the Instruction, Go cue, Movement onset, or Hold, each projected into the time series of instantaneous execution subspaces from the same session. Solid curves indicate classification accuracy averaged across 10-fold cross-validation; the surrounding shaded areas indicate ± 1 standard deviation from that average; and different colors represent the three sessions from the same monkey, with black being their average. Horizontal lines indicate the range of classification accuracies that would have been obtained had the instantaneous subspaces been chosen randomly, which we estimated for each set of trajectory segments by bootstrapping—projecting the trajectory segments into a randomly selected 3D space, training an LSTM decoder, and classifying single trials, repeated 500 times (Natraj et al., 2022).

As might have been expected based on the trajectory separations illustrated in Figure 3, classification accuracy peaked at a time point within or near the duration of the corresponding 100 millisecond trajectory segments, again indicating that the subspaces differed at times I, G, M, and H. Classification accuracy decreased progressively at times preceding and following each of these peaks. In monkey F (top row), for example, mean classification of the Instruction trajectory segments (Figure 4A) initially was close to 0.25, rose to 0.47 around the time of the instruction onset, and then fell back to 0.25. Mean accuracy for the Go segments (Figure 4B) began close to chance, rose gradually during the delay epoch to peak at 0.70 around the time of the Go cue, and decreased thereafter. For the Movement (Figure 4C) and Hold (Figure 4D) segments, classification accuracy started near 0.5 and peaked at 0.94 and 0.99 near the time of those events, respectively. Similar trends were seen for monkeys R (middle row) and T (bottom row). For each monkey classification accuracy for each of the four sets of trajectory segments—Instruction, Go, Movement, and Hold—as a function of time was quite consistent.

Although classification accuracy consistently peaked near the time of the behavioral event immediately after which each set of trajectory segments was sampled, the decline in accuracy before and after the peak differed depending on the behavioral event. Peak classification accuracy for Instruction segments was modest, beginning to rise from mean chance levels ~100 ms before the instruction onset and quickly falling back thereafter. Note that the early rise indicates that towards the end of the initial hold epoch, the PM mirror neuron subspace already was shifting in anticipation of the appearance of the instruction. At times outside of this brief peak, however, the instantaneous subspace was no more similar to that at the time of instruction onset than could be expected from chance alone.

In contrast, classification accuracy of the Go trajectory segments was elevated above mean chance levels for more of the RGM trial duration. Though exceeding 3 standard deviations from mean chance only late in the delay epoch, Go classification accuracy rose steadily through the delay epoch, peaked near the go cue, then fell back to near mean chance levels during the reaction (time G to M) and movement (time M to H) epochs. In monkeys F and R this rise began during the 500 ms instruction epoch, but only exceeded chance upper bound shortly before the Go cue. In monkey T, however, classification accuracy of the Go segments increased abruptly after the instruction onset, exceeding upper bound, and then continuing to increase through the instruction and delay epochs. We infer that as the instruction and delay epochs progressed, the instantaneous subspace thus shifted gradually, becoming progressively closer to that at the time of the Go cue.

Similarly, classification accuracy for the Movement trajectory segments increased gradually through the delay, and reaction epochs (G - M), peaking near the time of movement onset and then decreasing during the movement epoch (M – H). In monkey T, an abrupt increase occurred shortly after instruction onset, though classification accuracy did not exceed chance upper bound until after the Go cue. By the time of the Go cue, however, the instantaneous subspace already was close to that at the time of Movement onset.

Classification accuracy of the Hold trajectory segments increased relatively late in execution trials. During the delay and reaction epochs the instantaneous subspaces were no more similar than chance to that at the beginning of the hold epoch. Classification accuracy of the Hold trajectory segments began to increase only after Movement onset, rising through the movement epoch, peaking near the beginning of the hold epoch and decreasing thereafter.

To summarize, the instantaneous subspace of PM mirror neurons evoked by the appearance of the instruction was relatively short-lived, and not similar to the instantaneous subspaces later in execution trials. In contrast, as the instruction and delay epochs proceeded, the instantaneous subspace became increasingly similar to those found at the time of the Go cue and then movement onset. And then as the movement epoch proceeded, the instantaneous subspace became increasingly similar to that at the beginning of the final hold. Overall, the most prominent patterns of co-modulation among PM mirror neurons, represented by their instantaneous state-space, shifted continually as execution of RGM trials progressed.

### Instantaneous subspaces also shift progressively during observation

**Figure 5.**
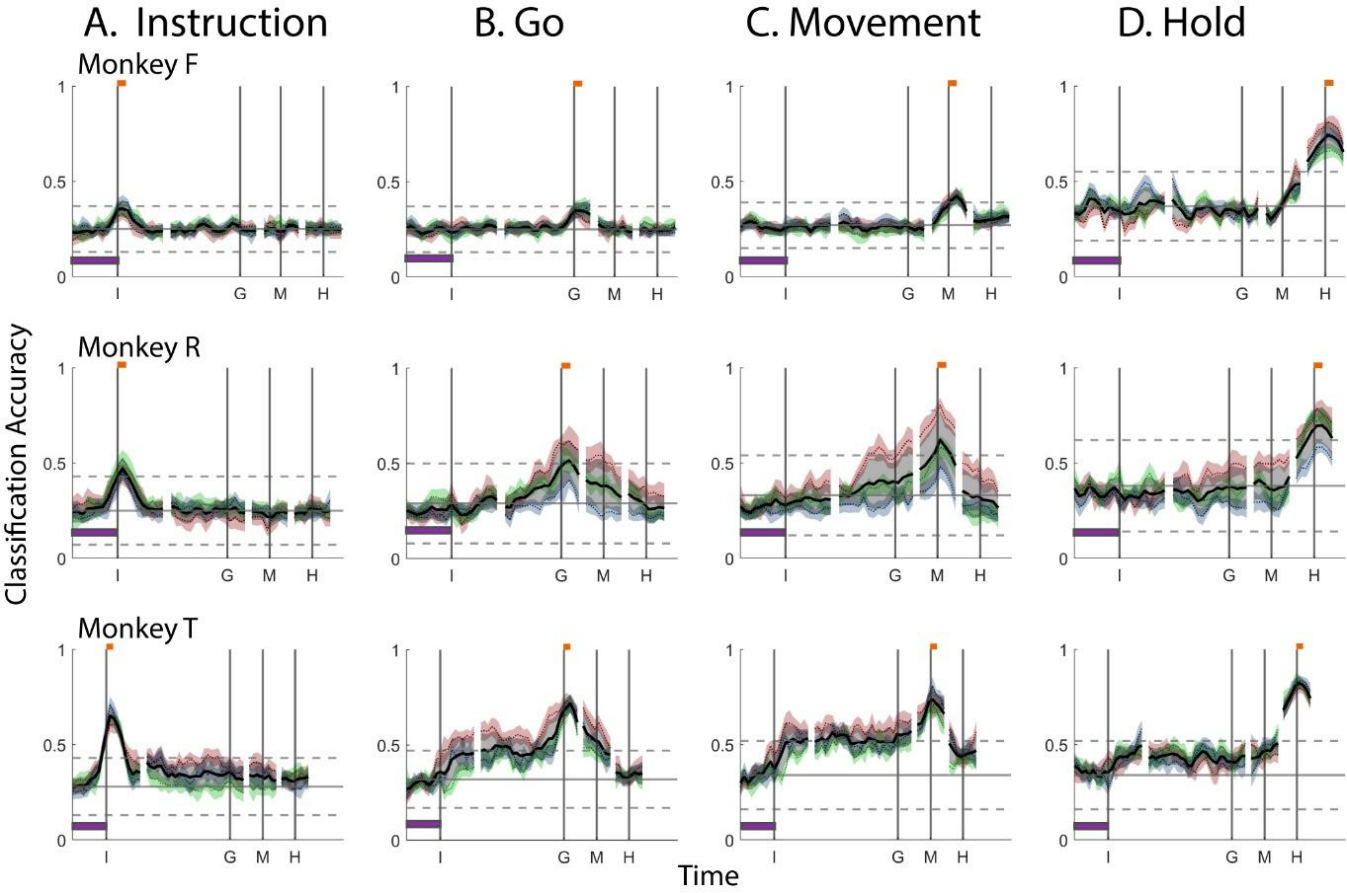
Classification accuracy for *observation trajectory segments* projected into instantaneous *observation subspaces*. **A.** Instruction trajectory segments. **B.** Go segments. **C.** Movement segments. **D.** Hold segments. The format of each frame is the same as that described for Figure 4.

Did the instantaneous PM mirror neuron subspace also shift continually during observation trials? We projected Instruction, Go, Movement, and Hold trajectory segments from observation trials into the time series of instantaneous subspaces identified for observation trials, again quantifying classification accuracy at each time point using the same approach above. Classification accuracy as a function of time during observation trials is shown in Figure 5.

For the Instruction trajectory segments, the brief peak of classification accuracy occurring around the time of instruction onset (I) during observation trials was quite like that found during execution trials. For the Go cue, Movement onset, and final Hold segments, however, classification accuracy tended to be lower, and the peaks near the time of these behavioral events (G, M, and H) tended to be comparatively short lived, particularly in monkey F.

In monkeys R and T, however, during observation as during execution, classification accuracy of the Go and Movement trajectory segments rose through the delay epoch (in monkey T again beginning abruptly after instruction onset), peaked near the time of the go cue (G) or movement onset (M), respectively, and then fell back to near chance levels during the reaction and movement epochs. In all three monkeys, classification accuracy of the Hold trajectory segments, during observation as during execution, began to increase from mean chance levels only after movement onset, rising through the movement epoch, peaking near the beginning of the hold epoch and decreasing thereafter. During observation, the progressive shifts in the instantaneous subspace from instruction, to go cue, to movement onset, to hold, though smaller, were similar to that found during execution.

### Do PM mirror neurons progress through the same subspaces during execution and observation?

**Figure 6.**
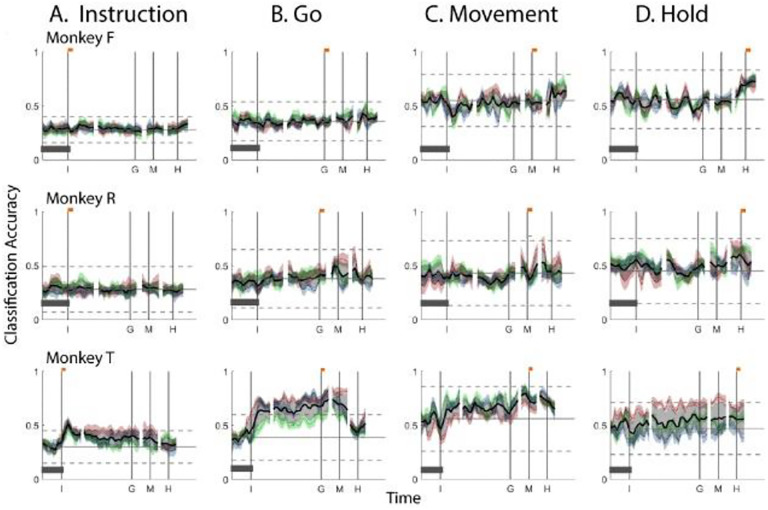
Classification accuracy for *execution trajectory segments* projected into instantaneous *observation subspaces*. **A.** Instruction trajectory segments. **B.** Go segments. **C.** Movement segments. **D.** Hold segments. The format of each frame is the same as that described for Figure 4.

**Figure 7.**
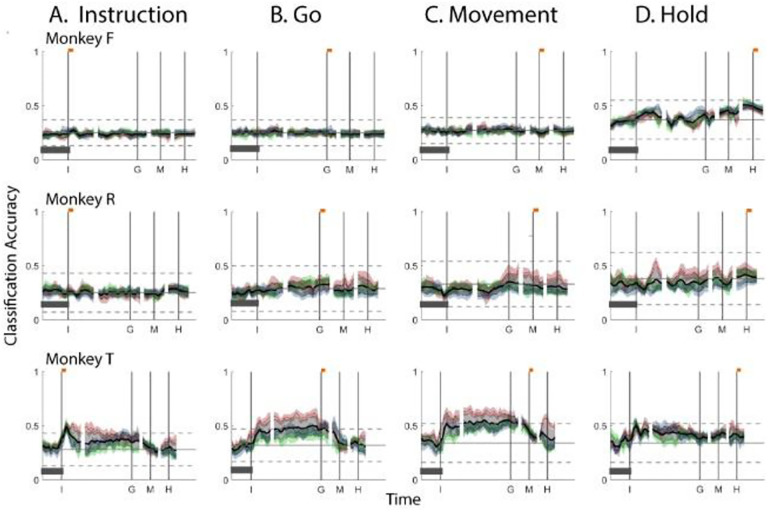
Classification accuracy for *observation trajectory segments* projected into instantaneous *execution subspaces*. **A.** Instruction trajectory segments. **B.** Go segments. **C.** Movement segments. **D.** Hold segments. The format of each frame is the same as that described for Figure 4.

Having found that PM mirror neuron populations show similar progressive shifts in their instantaneous neural subspace during execution and observation of RGM trials, we asked whether this continual progression passes through the same or similar instantaneous subspaces. To address this question, we cross-projected the Instruction, Go, Movement, and Hold trajectory segments from execution trials into the time series of instantaneous subspaces from observation trials (Figure 6), and cross-projected the trajectory segments from observation trials into the instantaneous subspaces from execution trials (Figure 7), again quantifying classification accuracy with separate LSTM decoders and 10-fold cross validation.

In neither monkey F or monkey R did either of these cross-projections show peaks of classification accuracy near the times of the behavioral events at which the trajectory segments were taken, as were previously seen in Figure 4 and Figure 5. Furthermore, at no time point did the classification accuracy in either cross-projection of the Instruction, Go, Movement, or Hold trajectory segments in either monkey F or monkey R exceed that expected from chance alone.

Only in monkey T did these cross-projections provide some classification accuracy beyond that expected from chance alone. In both cross-projections, the Instruction trajectory segments showed a brief peak shortly after instruction onset which was smaller (~0.5) than the corresponding peak found with either self-projection (~0.7). In both cross-projections, classification accuracy of both the Go and Movement segments rose shortly after instruction onset and remained elevated through the instruction and delay epochs, though exceeding chance only inconsistently. At no time point did the classification accuracy in either cross-projection of the Hold trajectory segments exceed that expected from chance alone upper bound. In neither cross-projection for monkey T did classification accuracy of the Go, Movement, or Hold trajectory segments show a peak near the time of the corresponding behavioral event. We infer that, although in monkey T the instantaneous subspaces through which PM mirror neuron population trajectories shifted showed some degree of similarity during execution and observation trials, in general, PM mirror neurons progressively shifted through different subspaces during execution versus observation.

### Execution-observation trajectory alignment

Having found little evidence that execution trajectory segments have decodable projections in instantaneous observation subspaces or vice versa, we asked whether execution and observation trajectory segments, each in their own instantaneous subspace, could be aligned. Such alignment would indicate that neural representations of trials involving the four objects were similarly related to one another during execution and observation, even though they occurred in different subspaces. For example, even though the set of recorded neurons changes from day to day altering the neural state space, the neural trajectories obtained from recordings made on different days during the same during center-out reaching movements can be aligned, indicating corresponding neural representations on both days (Gallego et al., 2020).

**Figure 8.**
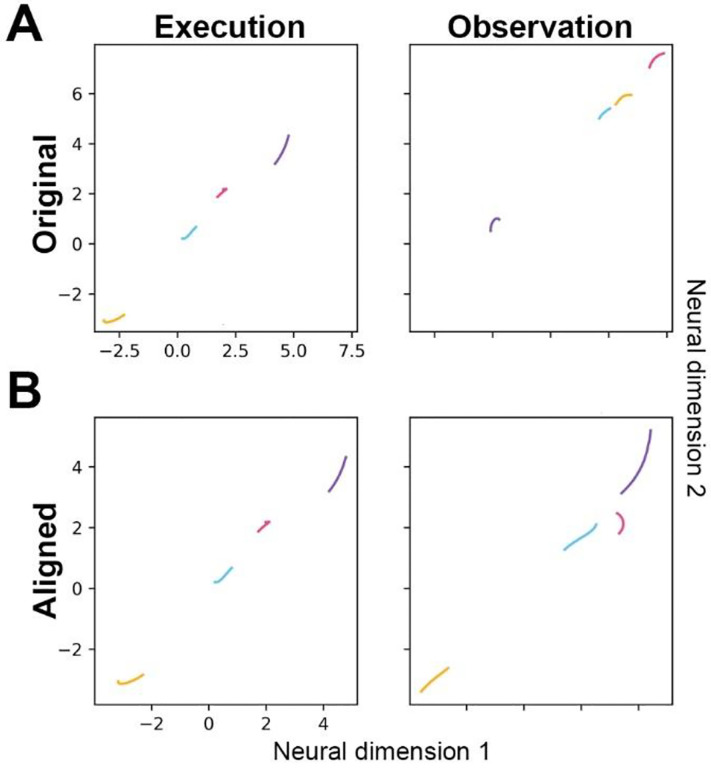
Alignment of execution and observation trajectory segments. **A.** Hold trajectory segments from execution trials are shown in the original instantaneous execution subspace at time H (left), and from observation trials in the original instantaneous observation subspace also at time H (right). **B.** After alignment, both execution (left) and observation (right) segments have been projection into the original execution subspace. Colors indicate trajectory segments from trials involving the sphere – purple, button – cyan, coaxial cylinder – magenta, perpendicular cylinder – yellow.

We therefore applied canonical correlation analysis (CCA, see Methods) to align the trajectories of observation trials with those of execution trials in the same recording session. For example, trial-averaged Hold trajectory segments in their original execution and observation subspaces before alignment are shown in Figure 8A, and after alignment in Figure 8B. In the original execution subspace, the order of the trajectory segments from lower left to upper right is yellow-cyan-magenta-purple, whereas the order in the observation subspace is purple-cyan-yellow-magenta. But after alignment, both execution and observation trajectories are ordered yellow-cyan-magenta-purple.

We aligned the Instruction trajectory segments from execution and observation trials and used a bootstrapping approach to evaluate the variability of this alignment. For each of 500 iterations, we randomly selected with replacement 20 execution trials and 20 observation trials involving each of the 4 objects. We projected these Instruction trajectory segments into the instantaneous subspace that provided the maximal LSTM classification accuracy using all execution or all observation trials, respectively. We then applied CCA to align the trajectories of those 80 execution trials and 80 observation trials. This process was repeated separately for Go, Movement, and Hold segments.

**Figure 9.**
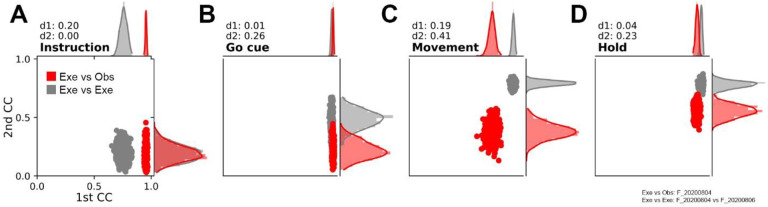
Alignment of latent dynamics (trajectory segments). **A.** Instruction trajectory segments. **B.** Go segments. **C.** Movement segments. **D.** Hold segments. Two-dimensional and marginal distributions are shown of the 1^st^ versus 2^nd^ correlation coefficients from 500 repetitions of aligning each set of trajectory segments separately. Values for alignment of execution and observation segments from the same session (F_20200804) are shown in red; values for alignment of execution segments from two sessions two days apart (F_20200804, F_20200806) are shown in gray. Wasserstein distances between the marginal distributions in the 1^st^ and 2^nd^ dimensions are given as d1 and d2 at the upper left of each plot.

The red scatter plots and marginal histograms in Figure 9 illustrate the resulting distributions of bootstrapped correlation coefficients in the two CCA dimensions for one session. The aligned Instruction and Go trajectory segments were highly correlated in the 1^st^ CCA dimension (mean bootstrapped r = 0.95 and 0.96, respectively), but not in the 2^nd^ CCA dimension (mean bootstrapped r = 0.19 and 0.22, respectively). The Movement and Hold trajectory segments showed slightly less correlation in the 1^st^ dimension (mean bootstrapped r = 0.73 and 0.89, respectively), but increased correlation in the 2^nd^ dimension (mean bootstrapped r = 0.38 and 0.55, respectively). Overall, the alignment of the execution and observation trajectories thus increased for the Movement and Hold segments as compared to the Instruction and Go segments, becoming highest for the Hold segments.

For comparison, we performed similar alignment of trajectory using execution trials from two different sessions from the same monkey recorded 2 days apart. The gray scatter plots and marginal histograms in Figure 9 illustrate the results of this execution-execution alignment. The aligned Instruction trajectory segments were moderately correlated in the 1^st^ CCA dimension (mean bootstrapped r = 0.76), but not in the 2^nd^ CCA dimension (mean bootstrapped r = 0.19).

The Go, Movement and Hold trajectory segments were highly correlations in the 1^st^ CCA dimension (mean bootstrapped r = 0.95, 0.91, and 0.93, respectively) and also showed increased correlations in the 2^nd^ CCA dimension (mean bootstrapped r = 0.48, 0.79, and 0.78 respectively). Execution-execution alignment thus was no better than execution-observation alignment at the time of Instruction onset, but increased progressively thereafter, becoming greatest around the time of movement onset. At the time of instruction onset, execution-execution and execution-observation alignments were comparable, but the execution-execution alignment outcompeted execution-observation one gradually with the most prominent difference at movement epoch, which we termed ‘dynamical similarity’.

To quantify these differences in each CCA dimension, we calculated the Wasserstein distance (WSD, see Methods) between the marginal distributions. WSD values for the two dimensions (d1, d2) are given in the upper left corner of each panel in Figure 9. In this example, execution and observation neural trajectories from the same session (red), while showing better alignment for Movement and Hold than for Instruction and Go cue segments, did not align quite as well as execution neural trajectories from two different sessions.

To determine whether the examples of Figure 9 were representative across the sessions from all three monkeys we compiled the correlation and WSD values across all sessions. Table 4 presents the average ± standard deviation for each mean bootstrapped correlation coefficient and WSD average across all sessions.

For execution-observation alignments within sessions CC1 did not vary significantly among the four trajectory segments (p = 0.16, h = 5.08, Kruskal-Wallis test), whereas CC2 did (p = 0.0035, h = 13.58, Kruskal-Wallis test). Post-hoc testing showed that CC2 was higher for Movement than Instruction trajectory segments, and higher for Hold than for either Instruction or Go segments (Dunn tests: M – I, p = 0.032; H – I, p = 0.0005; H – G, p = 0.014). Execution-observation alignment thus increased for trajectory segments later in the trials becoming highest for the Hold segments.

As for execution-observation alignments, CC1 for execution-execution alignments did not vary among the four trajectory segments (p = 0.15, h = 5.27, Kruskal-Wallis tests), whereas CC2 did (p = 5.30e-05, h = 22.4, Kruskal-Wallis test). Post-hoc testing showed that CC2 was higher for the Movement trajectory segments than for the Instruction or Go segments and higher for the Hold segments than for the Instruction segments (Dunn tests: M – I, p = 0.00001; M – G, p = 0.004; H – I, p = 0.001). While showing higher CC2 values than execution-observation alignment, execution-execution alignment also increased for trajectory segments later in the trial. But whereas execution-observation alignment was greatest for the Hold, execution-execution alignment was greatest for the Movement trajectory segments.

The WSDs for CC1 did not vary among the Instruction, Go, Movement, and Hold trajectory segments (p = 0.073, h = 6.96, Kruskal-Wallis test), whereas the WSDs for CC2 did (p = 0.00015, h = 20.3, Kruskal-Wallis test). Post-hoc testing showed that the WSDs for CC2 differed for all pairwise combinations of segments except G – H (Dunn tests, p < 0.05). The quantitative difference in execution-observation versus execution-execution alignment thus increased as trials progressed, becoming greatest for the Movement segments.

In summary, CCA showed that the neural trajectory segments during execution and observation were less well aligned than trajectory segments during two execution sessions. However, the alignment of neural representations during execution and observation, like that during two execution sessions, increased as behavioral trials progressed.

## DISCUSSION

We developed a novel approach to examine the progressive shifting of the neural state over the time course of behavioral trials involving reach, grasp, and manipulation. Rather than examining neural trajectories in fixed subspaces that captured their entire time course, we sampled brief (100 ms) segments of the neural trajectories at the times of four well-defined behavioral events and then projected these trajectory segments into each of a time series of instantaneous subspaces.

Using this approach, we found that PM MN populations recruit progressively shifting subspaces as monkeys both execute and observe RGM movements. The progressive shifting of the instantaneous subspace found here during execution trials resembles that found previously using fractional overlap of condition-dependent variance in M1 neuron populations (Rouse and Schieber, 2018). Although the progressive shifting described here is equivalent to progressive *rotation of the subspace*, we use the word ‘shift’ to contrast with the *rotation of the neural trajectory* in a fixed subspace described in other studies, particularly those using jPCA (Churchland et al., 2012; Russo et al., 2020).

Here, we found that progressive shifting of the condition-dependent subspace of PM MNs occurred as well when the monkey observed RGM trials performed by an experimenter. The instantaneous subspaces during execution and during observation were distinct, however. Yet despite this difference, execution and observation trajectory segments could be aligned, particularly the Movement and Hold segments, indicating corresponding representations of the four different RGM movements in the two behavioral contexts.

Corresponding representations of action execution and observation during task epochs with higher neural firing rates have been found previously in PMd MNs and in PMv MNs using representational similarity analysis (Papadourakis and Raos, 2019). Our approach, however, enabled a more continuous examination of the temporal evolution of this correspondence. We used the classification accuracy of LSTM decoding as a measure of the similarity of each instantaneous subspace to the subspace at the time trajectory segments were sampled. While the values obtained at most time points were less than 3 standard deviations from the mean of random subspaces, we nevertheless found the systematic temporal evolution of classification accuracy to be informative about the progressive shifting of the instantaneous subspace over the course of the trial.

### Features of subspace shifting during execution and observation

Projection of trajectory segments sampled in the 100 ms following instruction onset (at time I) showed a short-lived peak, reminiscent of the short bursts of “signal” discharge known to occur in a substantial fraction of PMd neurons following an instructional stimulus (Weinrich et al., 1984; Cisek and Kalaska, 2004). These peaks in each monkey were quite similar in their time course for execution and observation. They began to rise from their baseline ~100 ms before instruction onset, indicating that the instantaneous subspace already was shifting toward the subspace that could be anticipated at the time the instruction would appear. This early shift indicates that the anticipatory activity often seen in single neurons is not entirely non-specific (Mauritz and Wise, 1986), but rather represents a positioning of the neural population to receive the anticipated instruction. This peak declined rapidly after instruction onset, reaching baseline levels before the end of the Instruction epoch, except in monkey T where classification accuracy remained slightly above baseline through the delay and reaction epochs. The instantaneous subspace thus quickly shifted away from the Instruction subspace that was present just after instruction onset.

The firing rates of MNs in both PMv and PMd have been shown previously to modulate during preparatory delay periods in anticipation of a Go cue (Cisek and Kalaska, 2004; Maranesi et al., 2014). The present findings provide additional insight into this delay epoch activity. As the 500 ms instruction epoch ended, shifting of the subspace came to differ in execution versus observation contexts. During execution trials, the instantaneous subspace in all three monkeys began to shift toward both the subspace which would be present at time G and that which would be present at time M. This progressed steadily through the delay period. In monkey T, this progressive shift additionally was preceded by an abrupt shift in the subspace ~100 ms after instruction onset. This abrupt shift rendered the instantaneous subspace in monkey T partially similar to the I, G, and M subspaces simultaneously. After time G, the instantaneous subspace in each monkey shifted toward that at time M, and after time M, toward that at time H. In contrast to the subspaces at times G and M, the instantaneous subspace did not begin to shift toward that present at time H during the delay epoch, beginning instead until just before movement onset.

During observation trials, the shift of the instantaneous subspace toward that present at the time of the Instruction was quite similar to that found during execution trials. In contrast, the progressive shifts toward the Go, Movement, and Hold subspaces during observation were short-lived compared to those during execution. Nevertheless, these shifts began ~100 ms or more prior to each of the four events in all three monkeys, indicating that during observation the PM MN population predicted rather than responded to the behavior performed by the experimenter. Although in monkey T an abrupt shift toward the Go and Movement subspaces again occurred ~100 ms after instruction onset, progressive shifts toward these subspaces did not progress through the delay epoch, and instead began only shortly before each event, with progressive shifts away from each event subspace occurring as rapidly as away from the Instruction subspace. Likewise, the progressive shift of the instantaneous subspace toward the Hold subspace did not begin until after movement onset when the experimenter’s movement was already underway, though still predictive of the Hold per se.

During execution of a reaching task, condition-dependent subspaces are orthogonal during the preparatory delay versus movement epochs (Kaufman et al., 2014; Elsayed et al., 2016). In contrast, our findings suggest that in the present RGM task the condition-dependent subspace during the preparatory delay epoch was not entirely orthogonal to that during the movement epoch. Rather, the condition-dependent subspace shifted progressively closer to the Movement subspace as the delay and reaction epochs proceeded. This difference may reflect differences in reaching without grasping versus the present RGM movements. These previous studies, however, specifically identified preparatory and movement subspaces optimized to be orthogonal to one another, while the present approach did not.

### Distinct representations of execution and observation

Mirror neurons originally were thought to provide highly congruent neural representations of action execution and action observation. Our cross projections of execution trajectory segments into instantaneous observation subspaces and vice versa (Figures 6 & 7) indicate, however, that the condition-dependent subspaces traversed by PM MNs during the present RGM trials were distinct during execution and observation. The present findings are consistent with recent studies that have emphasized the considerable fraction of neurons with non-congruent activity in these two contexts, as well as differences in neural population activity during action execution versus action observation (Jiang et al., 2020; Pomper et al., 2023). As more situations have been investigated, the number of conditions needed to define a true mirror neuron in the strict sense of being entirely congruent has grown, making the duration of such congruence brief and/or its likelihood comparable to chance (Papadourakis and Raos, 2019; Pomper et al., 2023). True mirror neurons in the strictly congruent sense thus become something akin to grandmother cells (Bowers et al., 2019). While small numbers of such neurons might exist, their rarity precludes analysis of substantial populations, and we therefore have considered all neurons modulated significantly during both action execution and action observation as mirror neurons.

Two of the present monkeys, F and R, showed no appreciable cross projection of either execution trajectories into observation subspaces or observation trajectories into execution subspaces, indicating that the subspaces during execution and observation were orthogonal to one another. For both cross projections, however, monkey T’s PM MNs did show a brief peak in classification accuracy ~100 ms after instruction onset for cross-projected Instruction segments, and for cross-projected Go or Movement segments an abrupt increase in classification accuracy after instruction onset followed by a plateau sustained into the reaction epoch. No peaks were present, however, near the time of the go cue, movement onset or hold. Nevertheless, these findings suggest that the condition-dependent instantaneous subspaces in monkey T were shared partially during execution and observation. Although monkey T might be considered the outlier, we note that nearly twice as many PM MNs were recorded in monkey T as compared to monkey F or R. These findings in monkey T of a shared subspace containing part of the condition-dependent PM MN activity, with other activity in distinct (orthogonal) execution or observation subspaces are consistent with a recent study examining the subspaces containing the entire temporal trajectories of PMd/M1 MNs during center-out reaching movements (Jiang et al., 2020).

That neural representations of execution versus observation are distinct is not surprising given that the brain clearly is able to distinguish the same action performed in the two contexts. Nevertheless, we found that trajectory segments—which represent the predominant modes of co-modulation among PM MNs—could be aligned during execution and observation. The alignment between execution and observation segments from the same session was not as strong as alignment between execution segments from two different session. The weaker alignment of execution-observation trajectory segments may have resulted simply from the lower firing rates of PM MNs typically found during observation as compared to execution trials (Ferroni et al., 2021). Like execution-execution alignment, however, execution-observation alignment became stronger as trials progressed from Instruction, to Go, to Movement, to Hold, reflecting distinct neural representations for the two behavioral contexts with dynamical similarity.

### The role of mirror neuron populations

Although we did not track extraocular movements, video monitoring demonstrated that our monkeys remained attentive, actively scanning the visual environment throughout the blocks of observation trials. Though perhaps not following the experimenter’s movements closely with eye movements, the present results in and of themselves demonstrate that during observation trials the PM MN population was processing information on both the sequential epochs of the behavioral task (Mazurek et al., 2018), as well as the object to which the experimenter’s actions were directed on each trial. Moreover, ~100–200 ms before the Instruction onset, the Go cue, or Movement onset, when the experimenter had not yet begun to move, the instantaneous subspace of PM MNs began to shift toward the subspace that would be present at the time of each of those behavioral events. Though during observation such predictive activity is less intense and relatively short-lived compared to execution, these findings are consistent with the notion that the PM MN population predictively represents the sequence of behavioral events during observation trials (Kilner et al., 2007; Maranesi et al., 2014; Ferroni et al., 2021).

Yet if the representations of executed versus observed actions are distinct, how can the PM MN population nevertheless predictively represent that the same action is being performed by the subject and by another individual? Two features of population activity may provide such representation. First, the similar structure of the execution and observation population activity revealed by alignment of their latent dynamics through CCA may constitute a neural representation of the same action independent of the actor. Second, whereas the present analyses as well as others have focused on the condition-dependent variance in MN population activity (Jiang et al., 2020), still other studies that have not separated the condition-dependent versus condition-independent variance in neural activity have described more similar neural dynamics during execution and observation (Mazurek et al., 2018; Jerjian et al., 2020; Pezzulo et al., 2022). We speculate that while condition-dependent activity may represent particular movement types within a class of actions in a manner that differs depending on the actor, the condition-independent variance may provide a neural representation of a class of actions independent of the actor. Testing this hypothesis experimentally may require neural data from the same individuals performing and observing substantially different classes of actions.

## METHODS

Three Rhesus monkeys, F, R and T (an 11 kg male, a 6 kg female, and a 10 kg male, *Macaca mulatta*) were used in the present study. All procedures for the care and use of these non-human primates followed the Guide for the Care and Use of Laboratory Animals and were approved by the University Committee on Animal Resources at the University of Rochester, Rochester, New York.

### Execution trials

Each monkey was trained to perform a Reach-Grasp-Manipulate (RGM) task (Figure 2). Prior to each trial a ring of blue LEDs was illuminated around the pole supporting a center object and a 4 kHz tone began, both signaling the end of an inter-trial interval and the opportunity to begin a new trial. The monkey initiated the following sequence by pulling the center object for an initial hold epoch of variable duration (500–1000 ms). A ring of blue LEDs around the pole supporting one of four peripheral objects then was illuminated instructing the monkey as to the target object for the current trial. After 500 ms these instruction LEDs were extinguished, and the monkey was required to wait for a preparatory delay epoch lasting 500–2000 ms. At the end of this preparatory delay epoch, the blue LEDs for the center object were extinguished and the 4 kHz tone ceased, providing a “Go” cue. The monkey then reached to, grasped, and manipulated the remembered target object: turning a sphere, pushing a button, pulling a coaxial cylinder, or pulling a perpendicular cylinder. Once the instructed object had been manipulated, a ring of green LEDs around the object illuminated (indicating successful manipulation of the object) and the ring of blue LEDs for that object also illuminated (indicating correct object). The monkey then was required to hold the instructed object in its manipulated position for a final hold epoch of 1000 ms, after which the blue LEDs were extinguished. (The green LEDs extinguished whenever the monkey released the object.) After a 300 ms delay, the monkey received a liquid reward on each successful trial.

The selection and sequence of target objects in successive trials was controlled by custom software (Unified Task Control System, Gil Rivlis), which also 1) generated behavior event marker codes (Figure 2B), and 2) arranged trials involving the four different objects in a pseudorandom block design. The behavior event marker codes marked the times at which specific behavioral events occurred: Start of trial, Instruction onset, Instruction offset, Go cue (delay epoch ended), Movement onset, Hold began, Hold ended, End of trial. One trial involving each of the four different objects was presented sequentially in a block. Once a block had been completed, the sequence of the four objects was shuffled randomly for the next block. To prevent the monkey from skipping more difficult objects, if the monkey failed to complete a trial successfully the same target was repeated until the monkey succeeded.

### Observation trials

In a separate block of trials, the monkey observed an experimenter performing the same RGM task. The experimenter occasionally made errors intentionally. The monkey received a reward each time the experimenter performed a successful trial, but not when the experimenter made an error, which kept the monkey attentive to the experimenter’s performance. Although extraocular movements were not recorded or controlled, video monitoring verified that the monkey remained alert and attentive throughout blocks of observation trials.

### Neuron Recording

The three monkeys each were implanted with Floating Microelectrode Arrays (FMAs, Microprobes for Life Sciences), in the ventral premotor cortex (PMv) and in the dorsal premotor cortex (PMd). In monkey F, 32-channel FMAs were used; in monkeys R and T, 16-channel FMAs were used. Monkeys F and R each had a total of 64 recording electrodes implanted in PMd and 64 in PMv, whereas monkey T had 64 in PMd, but only 48 in PMv. Broadband signals were recorded simultaneously from all 128 electrodes using a Nomad/Trellis data acquisition system (Ripple, Salt Lake City, UT), which also recorded the behavioral event marker codes generated by the behavioral control system. In each recording session, data were collected during similar numbers of successful trials involving each target object during execution and then during observation, as summarized in Table 2. Off-line, spike waveforms were extracted and sorted using custom software. Sorted units were classified as definite single units, probably single units, multi-units, or noise based on their signal-to-noise ratio and estimated false-positive fraction using previously published criteria (Rouse and Schieber, 2016).

### Mirror Neuron Identification

Each definite single unit, probable single unit or multi-unit was tested for task-related modulation. Because a given neuron’s firing rates during execution and observation trials almost always differed (Ferroni et al., 2021; Pomper et al., 2023), we tested each unit for modulation using data from these two contexts separately. Spike counts from each successful behavioral trial were extracted during eleven 200 ms periods: i) before instruction onset, ii) after instruction onset, iii) before instruction offset, iv) after instruction offset (delay began), v) before delay ended, vi) after delay ended (reaction time began), vii) before movement onset, viii) after movement onset (movement time began), ix) before movement ended, x) after movement ended (final hold began), xi) hold ended. We then conducted two-way rmANOVAs on these spike counts using object and time period as factors. We considered a unit task-related if it showed a significant main effect of either i) object or ii) time period, or a significant iii) interaction effect. Any unit modulated significantly both during execution and during observation was considered to be a mirror neuron. Because each unit thus had six opportunities to show significance, we used a corrected significance criterion of p<0.0083 (=0.05/6).

### Data analysis

Spike times for each neuron were binned (bin width = 1 ms), smoothed with a Gaussian kernel (σ = 50 ms) and square-root transformed to render variance similar from low to high firing rates. The activity of each neuron was time-aligned to four behavior events and truncated before and after using the median delay, reaction, and movement times per object and per session as follows: i) instruction onset (I)—500 ms before, 500 ms after; ii) go cue (G)—median delay duration before, half the median reaction time after; iii) movement onset (M)—half the median reaction time before, 200 ms after; and iv) start of final hold (H)—200 ms before, 200 ms after. These four snippets of neural activity were concatenated for each trial. Neural activity then was stored as a three-dimensional tensor (*N* × *K* × *T*, where *N* is number of neurons, *K* the number of trials, and *T* the number of time points) for each of four target objects.

### Instantaneous subspace identification

Instantaneous neural subspaces were identified separately using principal component analysis (PCA) of trial-averaged neural activity for each of the four objects at each 50 ms time step. Because three dimensions capture all the variance of four points, three principal component dimensions defined each subspace. Each subspace can be considered a filter that provides a matrix, *W*, which can project high-dimensional neural activity into the low-dimensional subspace. Since subspaces favored different co-modulation patterns, they can be considered as a set of filters.

### Trajectory visualization and separation

We projected 100 ms segments of neural activity into each instantaneous subspace by multiplying the neural activity, *X*(*t*), by the transforming matrix for the *i*^*th*^ subspace, *W*_*i*_ ,which yielded low dimensional trajectories, *L*(*t*) = *X*(*t*)*W*_*t*_ (*t* ∈ *T*). This process was repeated for each instantaneous subspace in the time domain of interest (*t* ∈ *T*).

To quantify the separation between the four trial-averaged trajectories from trials involving different objects in a given instantaneous subspace, we calculated their “cumulative separation” (*CS*) as:

$$CS=\frac{1}{T}\sum_{t\in T}D(t)=\frac{1}{T}\sum_{t\in T}\sum_{i\neq j}d_{ij}(t)$$

where *d*_*ij*_(*t*) is the Euclidean distance between the *i*^*th*^ and *j*^*th*^ trajectories at time point *t*. We summed the 6 pairwise distances between the 4 trajectory segments across time points and normalized by the number of time points, *T*. The larger the *CS*, the greater the separation of the trajectory segments.

### Subspace Comparisons

As illustrated schematically in Figures 1B, the same segment of high-dimensional neural activity projected into different instantaneous subspaces can generate low-dimensional trajectories of varying separation. The degree of separation will depend on the similarity of the subspaces into which the neural activity is projected. To quantify this separation, we projected trajectory segments into time series of instantaneous subspaces and trained a separate long short-term memory (LSTM) classifier for each subspace in the time series. Using MATLAB’s Deep Learning Toolbox, each classifier was trained on 40% of the trials available (using equal numbers of trials involving each of the four objects). The remaining 60% of the available trials were decoded by the trained classifier. This process was repeated 10 times and the mean ± standard deviation fraction correct across the 10 folds was reported as the classification accuracy at that time. Classification accuracy was plotted as a function of trial time.

### Similarity of aligned dynamics

We used Canonical Correlation Alignment (CCA) to compare the similarity of latent dynamics (Gallego et al., 2018, 2020; Safaie et al., 2022). In brief, given latent dynamics in two original spaces, *L*_*A*_ and *L*_*B*_, CCA finds a linear transformation such that the “aligned” latent dynamics, $\tilde{L_{A}}$ and $\tilde{L_{B}}$, are maximumly correlated in each dimension of a common subspace. Larger canonical correlations (CCs) indicate a higher degree of similarity.

We calculated the CCs between execution and observation trials from the same session and compared them to the CCs between execution trials from two different sessions. We used a bootstrapping approach to assess the variability of the resulting correlation coefficients. We randomly selected 20 trials involving each target object (totaling 80 trials) in each data set with replacement, extracted data from each trial during one of four task intervals (instruction, go cue, movement, and hold), and performed CCA. With 500 iterations, we obtained a distribution of the CCs between the two data sets in each of two dimensions.

### Wasserstein distance

We used Wasserstein distance to quantify the distance between two bootstrapped CC distributions. The Wasserstein distance (WSD), sometimes called the “earth-mover’s distance” or the “optimal transport plan” quantifies the distance between two probability distributions, irrespective of their underlying statistics (Vallender, 1974). Intuitively, the two distributions A and B can be considered to be two mounds of dirt. Distribution A can be transformed into B by moving dirt, which requires work, i.e. the product of an incremental mass of the dirt and the distance that increment must be moved, summed across all increments. The Wasserstein distance between A and B is the minimum possible total work. We used the SciPy stats.wasserstein_distance function to compute the WSD between the execution-observation CC distribution and the execution-execution CC for each CCA dimension. WSD was calculated separately for Instruction, Go, Movement, and Hold trajectory segments.

## Figures and Tables

**Figure 10.**
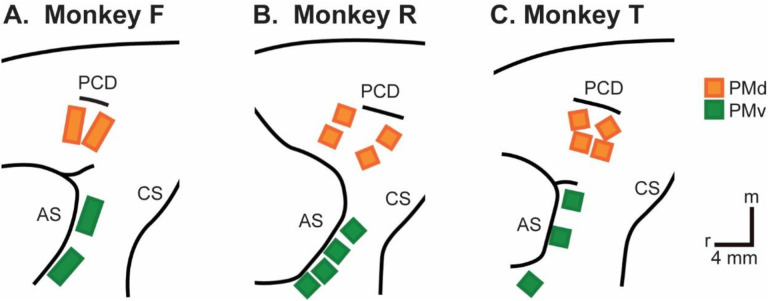
Recording array locations in **A.** Monkey F. **B.** Monkey R. **C.** Monkey T. PCD – precentral dimple; AS – arcuate sulcus; CS – central sulcus; r – rostral; m – medial. Scale bars apply to all three monkeys.

**Table 1.** Numbers of trials in each session

	Monkey F		Monkey R		Monkey T	
	Exe	Obs	Exe	Obs	Exe	Obs
Session 1	(58,59,62,63)	(71,72,71,72)	(22, 8,25,26)	(32,31,30,31)	(57,54,57,55)	(60,61,59,57)
Session 2	(59,58,60,56)	(73,72,75,74)	(34,26,34,38)	(40,41,40,37)	(47,53,52,43)	(57,53,58,58)
Session 3	(63,58,58,58)	(72,75,74,74)	(42,41,49,45)	(49,50,51,49)	(43,41,38,42)	(50,48,48,50)

For each of the three sessions from each of the three monkeys, numbers of trials involving each of the four objects (sphere, button, coaxial cylinder, perpendicular cylinder are given in parentheses separately for execution and for observation.

**Table 2.** Numbers of mirror neurons in each session

	Monkey F	Monkey R	Monkey T
Session 1	44 (24, 20)	48(19, 29)	79(37, 42)
Session 2	47 (32,15)	47(21, 26)	91(48, 43)
Session 3	42 (28,14)	37(19, 18)	100 (48, 52)

For each of the three sessions from each of the three monkeys, numbers of PM MNs are given in the format of Total(PMv, PMd).

**Table 3.** Alignment summary across sessions.

		Trajectory Segments			
		Instruction	Go	Movement	Hold
**Exe-Obs**	**CC1**	0.88 ± 0.11	0.89 ± 0.06	0.82 ± 0.09	0.84 ± 0.05
	**CC2**	0.28 ± 0.10	0.37 ± 0.15	0.48 ± 0.16	0.59 ± 0.08
**Exe-Exe**	**CC1**	0.84 ± 0.08	0.87 ± 0.08	0.94 ± 0.02	0.89 ± 0.04
	**CC2**	0.29 ± 0.10	0.53 ± 0.10	0.84 ± 0.08	0.73 ± 0.04
**WSD**	**d1**	0.07 ± 0.06	0.04 ± 0.04	0.12 ± 0.07	0.06 ± 0.05
	**d2**	0.03 ± 0.03	0.16 ± 0.06	0.36 ± 0.15	0.15 ± 0.07

For each set of trajectory segments, across-session means ± standard deviations are given for the 1^st^ and 2^nd^ alignment correlation coefficients (CC1, CC2), as well as for the WSDs (d1, d2) between the Exe-Obs and Exe-Exe bootstrapped marginal distributions.
