# Supplementary material for: Progressively shifting patterns of co-modulation among premotor cortex neurons carry dynamically similar signals during action execution and observation
Source: bioRxiv. 2025 Jul 10:2023.11.06.565833. Originally published 2023 Nov 6. Preprint. [Version 5] doi: 10.1101/2023.11.06.565833 (PMC10659317; doi:10.1101/2023.11.06.565833)
Supplement: 1 — Figure 4 – figure supplement 1. First, second, and third principal angles as a function of time. An example in which the three principal angles, θ1,θ2,θ3, between the instantaneous subspace at time M (movement onset) and the entire time series of instantaneous subspaces have been plotted as a function of time for PM MNs. (Data from monkey R, session 1.) Note that all three principal angles go to 0°at time M when the current instantaneous subspace is, by definition, the subspace at time M. Figure 4 – figure supplement 2. First principal angles between a fixed 3D subspace and 5000 other 3D subspaces randomly chosen from spaces of dimensionality, N, varying from 5 to 500. Error bars indicate ± 1 standard deviation from the mean. Note that as the dimensionality of the parent space decreases, the random principal angle also decreases. Figure 4 – figure supplement 3. Time course of the first principal angle of instantaneous subspaces for AE neurons during observation trials. As would be expected given that AE neurons were not modulated significantly during observation trials, in the observation context AE populations had no gradual changes in principal angle, showing only relatively sharp troughs dipping toward 0° at each of the four selected times when the current instantaneous subspace, by definition, approached that at times I, G, M, or H. Formatting is the same as in Figure 4. Figure 5 – figure supplement 1. Cumulative separation. To summarize the changes in trajectory separation illustrated in Figure 5, we calculated the 3-dimensional cumulative separation (CS, the summed pointwise Euclidean distance between all pairwise combinations of the four object-specific trajectory segments, see Methods) for each set of four segments projected into each of the four instantaneous subspaces at times I, G, M, or H. CS values, which we use only to characterize the phenomenon of trajectory separation, are illustrated for execution from the example session of Figure 5 as a color mat [file NIHPP2023.11.06.565833V5-supplement-1.pdf]

bioRxiv preprint doi: https://doi.org/10.1101/2023.11.06.565833; this version posted July 10, 2025. The copyright holder for this preprint (which was not certified by peer review) is the author/funder, who has granted bioRxiv a license to display the preprint in perpetuity. It is made available under aCC-BY 4.0 International license.

Figure Supplements

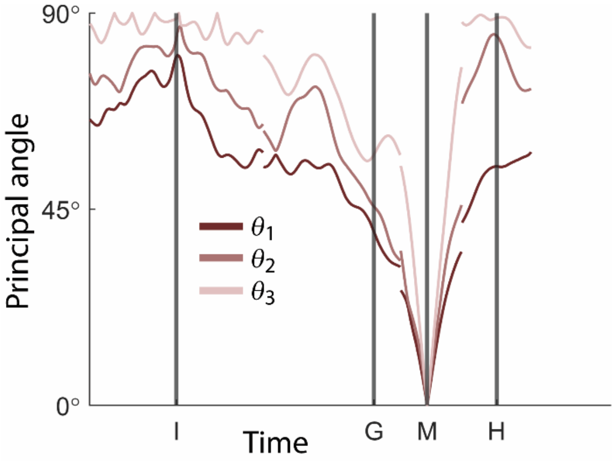

Figure 4 – figure supplement 1. First, second, and third principal angles as a function of time. An example in which the three principal angles, $\theta_1, \theta_2, \theta_3$, between the instantaneous subspace at time M (movement onset) and the entire time series of instantaneous subspaces have been plotted as a function of time for PM MNs. (Data from monkey R, session 1.) Note that all three principal angles go to 0°at time M when the current instantaneous subspace is, by definition, the subspace at time M.

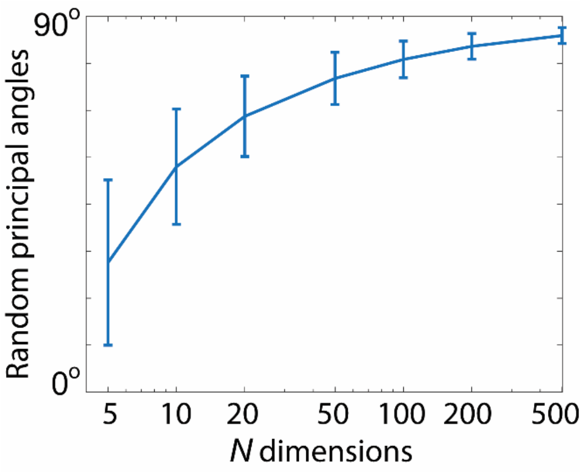

Figure 4 – figure supplement 2. First principal angles between a fixed 3D subspace and 5000 other 3D subspaces randomly chosen from spaces of dimensionality, *N*, varying from 5 to 500. Error bars indicate ± 1 standard deviation from the mean. Note that as the dimensionality of the parent space decreases, the random principal angle also decreases.

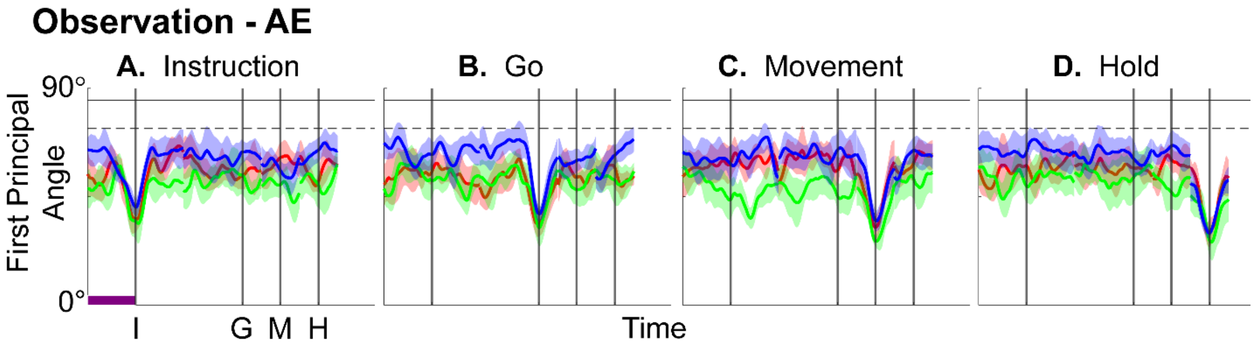

Figure 4 – figure supplement 3. Time course of the first principal angle of instantaneous subspaces for AE neurons during observation trials. As would be expected given that AE neurons were not modulated significantly during observation trials, in the observation context AE populations had no gradual changes in principal angle, showing only relatively sharp troughs dipping toward 0° at each of the four selected times when the current instantaneous subspace, by definition, approached that at times I, G, M, or H. Formatting is the same as in Figure 4.

bioRxiv preprint doi: https://doi.org/10.1101/2023.11.06.565833; this version posted July 10, 2025. The copyright holder for this preprint (which was not certified by peer review) is the author/funder, who has granted bioRxiv a license to display the preprint in perpetuity. It is made available under aCC-BY 4.0 International license.

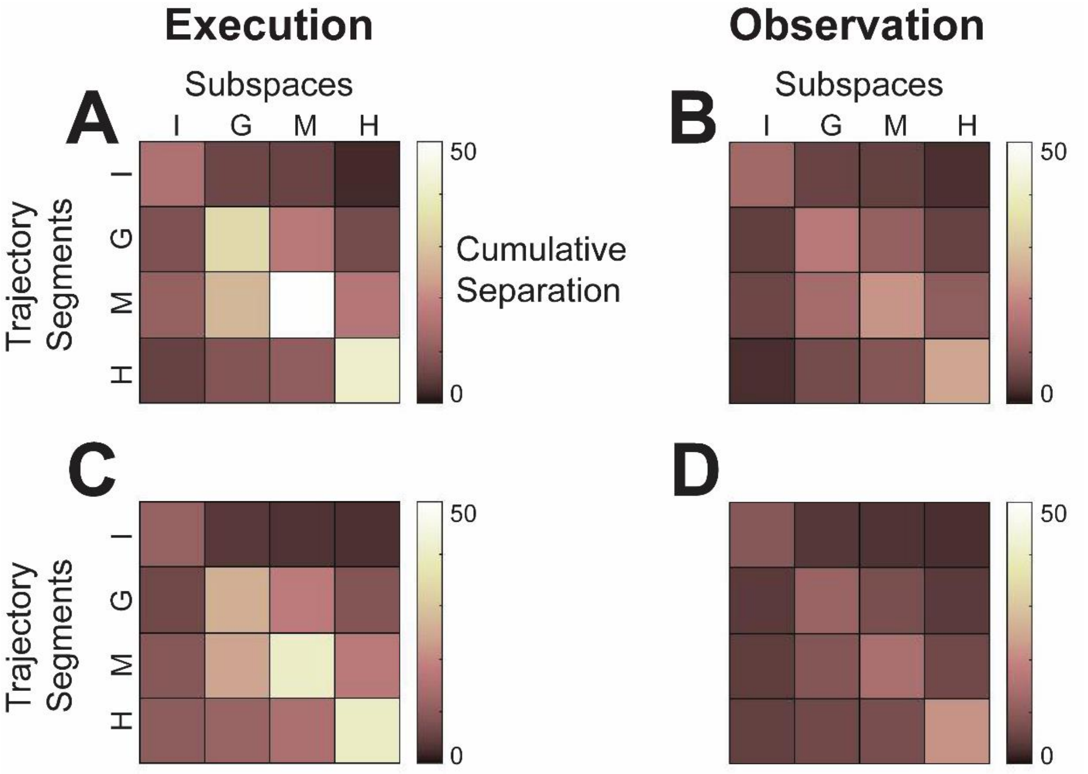

Figure 5 – figure supplement 1. Cumulative separation. To summarize the changes in trajectory separation illustrated in Figure 5, we calculated the 3-dimensional cumulative separation (C*S*, the summed pointwise Euclidean distance between all pairwise combinations of the four object-specific trajectory segments, see Methods) for each set of four segments projected into each of the four instantaneous subspaces at times I, G, M, or H. *CS* values, which we use only to characterize the phenomenon of trajectory separation, are illustrated for execution from the example session of Figure 5 as a color matrix in **A**, and for observation in **B**. For both execution and observation, the highest *CS* values lie on the main diagonal, increasing in temporal order from Instruction to Go to Movement to Hold, with the exception that for execution, *CS* for Hold was less than for Movement. **C** and **D** show *CS* matrices averaged across all three sessions from all three monkeys for execution and observation, respectively, demonstrating that the features seen in the example session of Figure 5 were relatively consistent across sessions. Across all nine sessions two-way ANOVA showed significant main effects on *CS* values of both segment and subspace as well as a significant interaction effect during both execution and observation ($p < 0.05$). In both of these contexts, as the instantaneous subspace of the PM MN population shifted progressively over the time course of RGM trials, the separation of condition-dependent neural trajectories also increased.

bioRxiv preprint doi: https://doi.org/10.1101/2023.11.06.565833; this version posted July 10, 2025. The copyright holder for this preprint (which was not certified by peer review) is the author/funder, who has granted bioRxiv a license to display the preprint in perpetuity. It is made available under aCC-BY 4.0 International license.

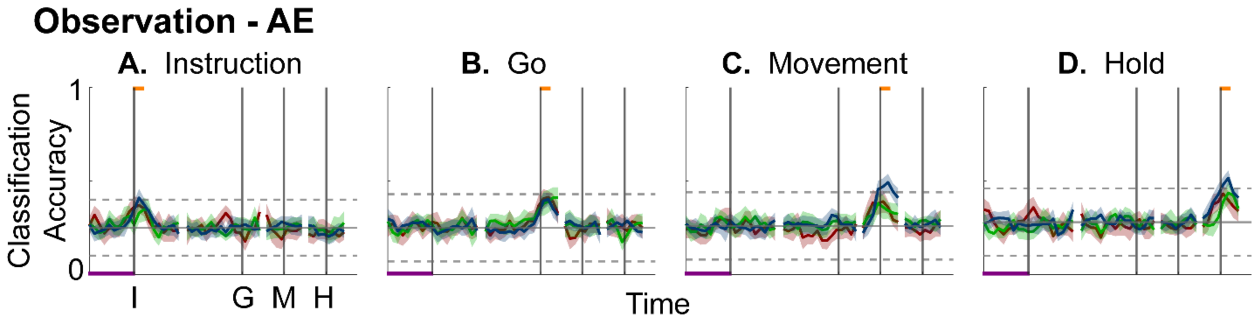

Figure 6 – figure supplement 1. Decodable information as a function of time in PM AE neuron populations. Formatting is the same as in Figure 6. As might have been expected, AE populations showed little if any decodable information during observation.

bioRxiv preprint doi: https://doi.org/10.1101/2023.11.06.565833; this version posted July 10, 2025. The copyright holder for this preprint (which was not certified by peer review) is the author/funder, who has granted bioRxiv a license to display the preprint in perpetuity. It is made available under aCC-BY 4.0 International license.

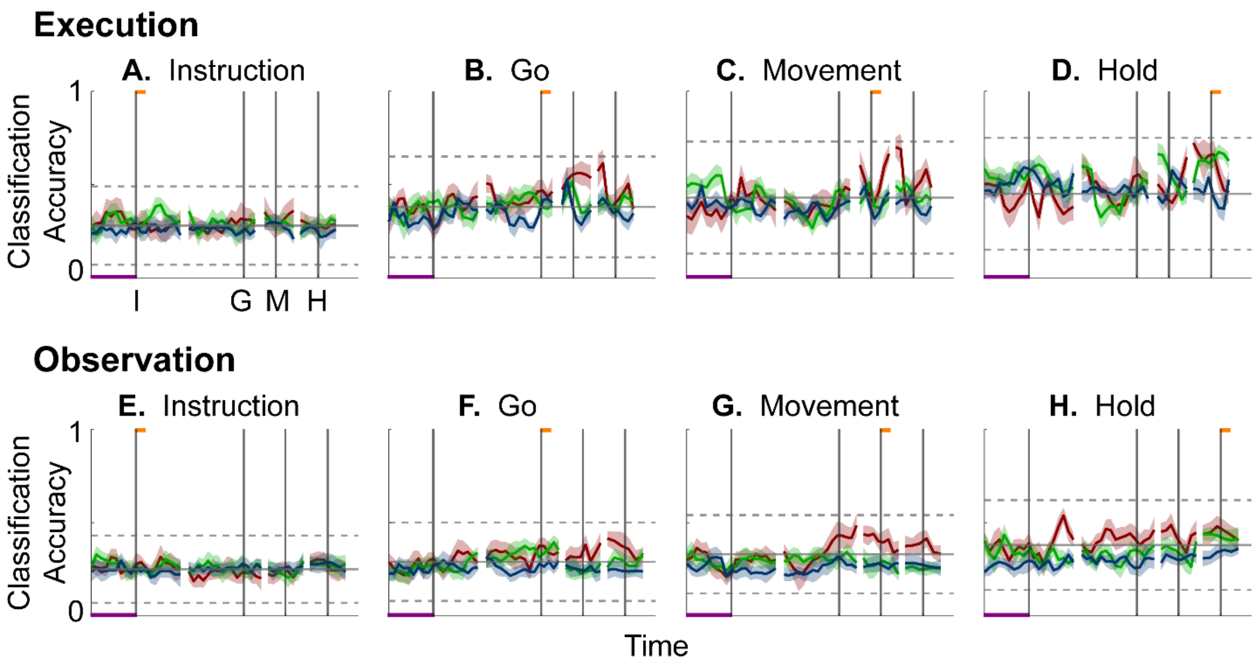

Figure 7 – figure supplement 1. Classification accuracy of trajectory segments cross-projected between instantaneous execution and observation subspaces of PM MNs as a function of time. On top, Instruction, Go, Movement, and Hold *execution trajectory segments* (**A, B, C, D,** respectively) from individual trials have projected into the time series of *instantaneous observation subspaces* and classified with a separate LSTM decoder at each time point; below, Instruction, Go, Movement, and Hold *observation trajectory segments* (**E, F, G, H,** respectively) have been projected into the time series of *instantaneous execution subspaces* and classified. Neither of these cross-projections showed gradual progression to peaks of classification accuracy. Nor did the classification accuracy in either cross-projection exceed that expected from chance alone (horizontal dashed lines). These results confirm that little if any overlap between instantaneous, condition-dependent execution and observation subspaces was present in monkey R. Findings were similar in monkey F. Formatting is the same as in Figure 6.

bioRxiv preprint doi: https://doi.org/10.1101/2023.11.06.565833; this version posted July 10, 2025. The copyright holder for this preprint (which was not certified by peer review) is the author/funder, who has granted bioRxiv a license to display the preprint in perpetuity. It is made available under aCC-BY 4.0 International license.

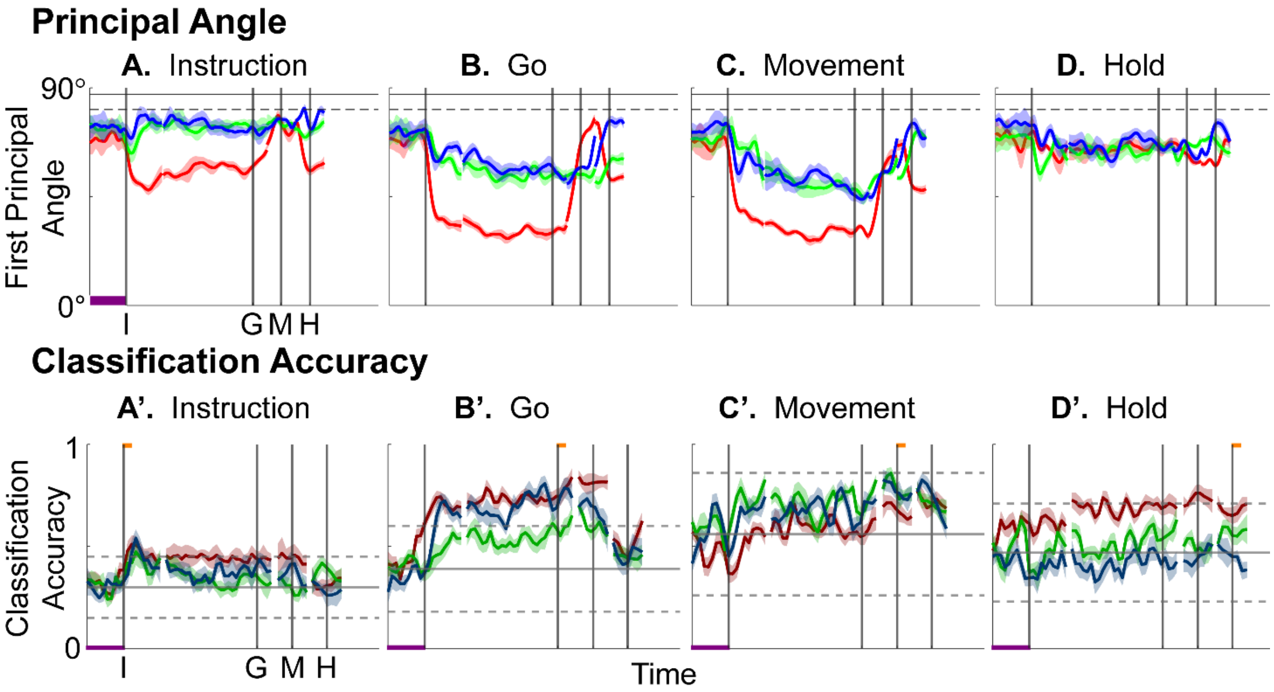

Figure 7 – figure supplement 2. Partial overlap of execution and observation subspaces in monkey T. **A-D**. The first principal angles between the instantaneous *execution* subspace at times I, G, or M (though not H) and the time series of instantaneous *observation* subspaces showed an abrupt drop beginning at the time of instruction onset (I) and continuing until the time of movement onset (M). This drop, which reflects partial overlap of the execution and observation subspaces, was marked during session 1 (red), but less so during sessions 2 and 3 (green and blue, respectively). **A'-D'**. Likewise, Instruction, Go, or Movement, *execution trajectory segments* projected into the time series of *instantaneous observation subspaces* showed a rise in decodable information, also indicative of some degree of overlap, beginning at the time of instruction onset (I). **A-D** are formatted as Figure 4; **A'-D'** as in Figure 6. Overlap like that seen here in monkey T was not found in monkeys R or F.
