# Supplementary material for: Progressively shifting patterns of co-modulation among premotor cortex neurons carry dynamically similar signals during action execution and observation
Source: bioRxiv. 2024 Nov 7:2023.11.06.565833. Originally published 2023 Nov 6. Preprint. [Version 3] doi: 10.1101/2023.11.06.565833 (PMC10659317; doi:10.1101/2023.11.06.565833)
Supplement: Supplementary file 1 [file NIHPP2023.11.06.565833V3-supplement-1.pdf]

bioRxiv preprint doi: https://doi.org/10.1101/2023.11.06.565833; this version posted November 8, 2024. The copyright holder for this preprint (which was not certified by peer review) is the author/funder, who has granted bioRxiv a license to display the preprint in perpetuity. It is made available under aCC-BY 4.0 International license.

SUPPLEMENTARY INFORMATION

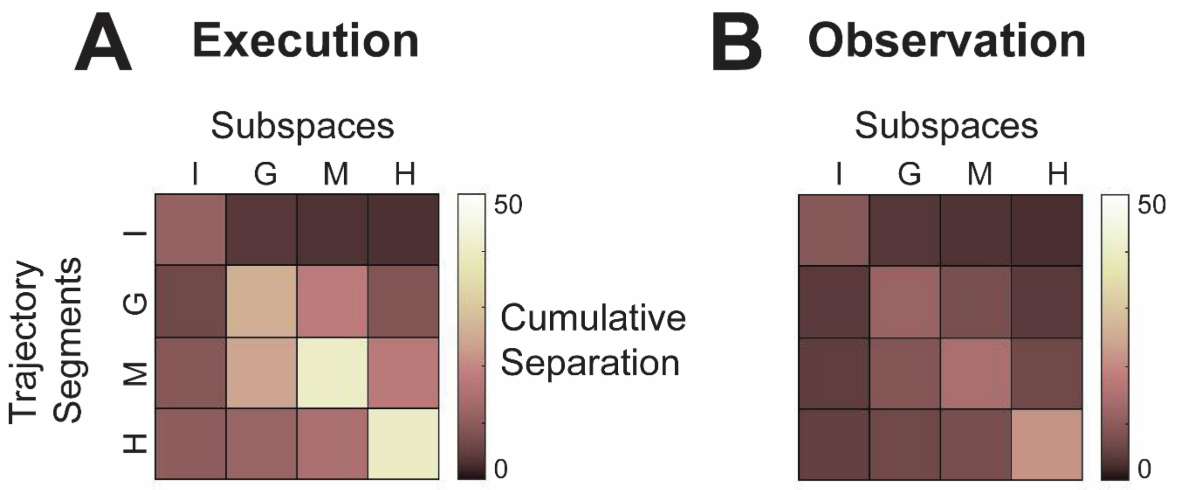

Supplementary Figure 1. *CS* values averaged across all 9 sessions from all 3 monkeys are shown as color matrices separately for **A.** Execution and **B.** Observation, illustrating that the same features shown for the single-session example of Figure 6 apply across all sessions.

bioRxiv preprint doi: https://doi.org/10.1101/2023.11.06.565833; this version posted November 8, 2024. The copyright holder for this preprint (which was not certified by peer review) is the author/funder, who has granted bioRxiv a license to display the preprint in perpetuity. It is made available under aCC-BY 4.0 International license.

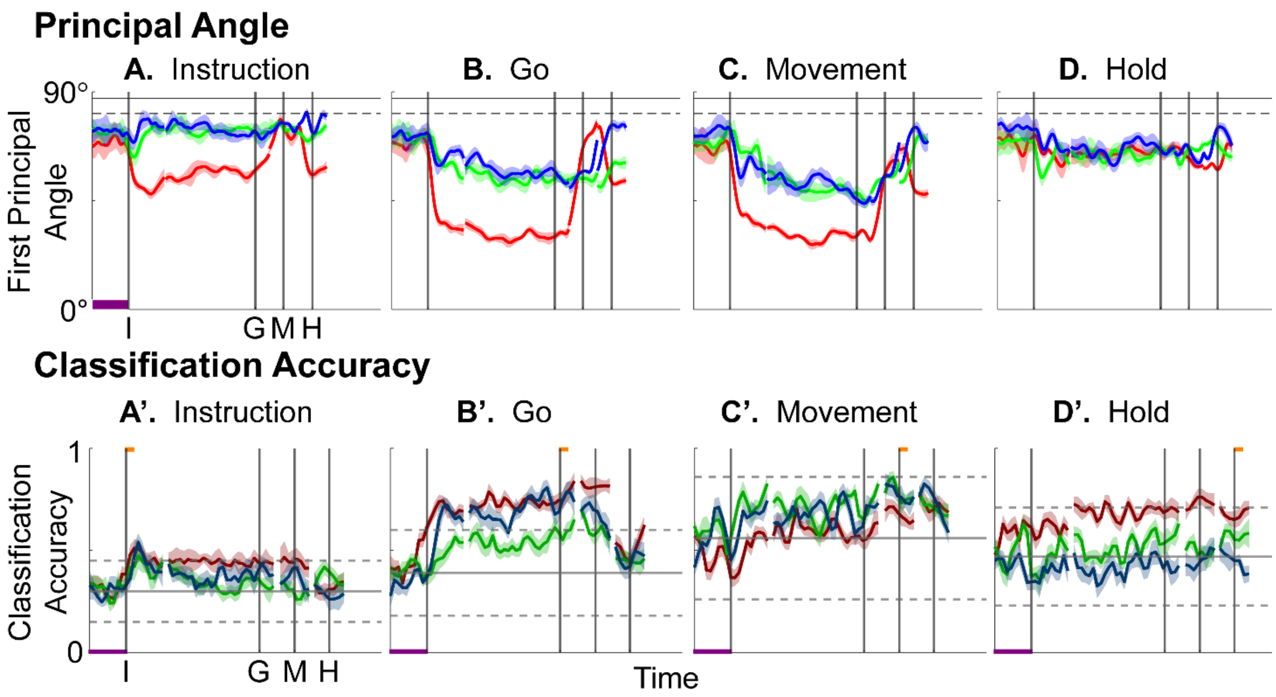

Supplementary Figure 2. Overlap of execution and observation subspaces in monkey T. In regard to overlap of instantaneous execution and observation subspaces, monkey T was an exception. **A-D**. The first principal angles between the instantaneous *execution* subspace at times I, G, or M (though not H) and the time series of instantaneous *observation* subspaces showed an abrupt drop beginning at the time of instruction onset (I) and continuing until the time of movement onset (M), most marked during session 1 (red). **A'-D'**. Likewise, the Instruction, Go, or Movement, *execution trajectory segments* projected into the time series of *instantaneous observation subspaces* showed a rise in decodable information beginning at the time of instruction onset (I). **A-D** formatted as Figure 9; **A'-D'** as in Figure 10.
